# Novel Perspectives on Food-Based Natural Antimicrobials: A Review of Recent Findings Published since 2020

**DOI:** 10.3390/microorganisms11092234

**Published:** 2023-09-05

**Authors:** Taner Sar, Pelin Kiraz, Vjola Braho, Sharareh Harirchi, Meltem Yesilcimen Akbas

**Affiliations:** 1 Swedish Centre for Resource Recovery, University of Borås, 50190 Borås, Sweden; vrrapaj@yahoo.com (V.B.); sharareh_harirchi@yahoo.com (S.H.); 2 Department of Molecular Biology and Genetics, Gebze Technical University, Gebze-Kocaeli 41400, Türkiye; pkiraz@gtu.edu.tr (P.K.); akbasm@gtu.edu.tr (M.Y.A.)

**Keywords:** extracts, essential oils, herbs, antibacterial activities, biotechnology, pathogens, food control

## Abstract

Various fruit and vegetable wastes, particularly peels, seeds, pulp, and unprocessed residues from the food industry, are abundant sources of antioxidants and essential antimicrobial agents. These valuable bioactive compounds recovered from the food industry have a great application in food, agriculture, medicine, and pharmacology. Food-derived natural antimicrobials offer advantages such as diminishing microbial loads and prolonging the shelf life of food products particularly prone to microbial spoilage. They not only enrich the foods with antioxidants but also help prevent microbial contamination, thereby prolonging their shelf life. Similarly, incorporating these natural antimicrobials into food packaging products extends the shelf life of meat products. Moreover, in agricultural practices, these natural antimicrobials act as eco-friendly pesticides, eliminating phytopathogenic microbes responsible for causing plant diseases. In medicine and pharmacology, they are being explored as potential therapeutic agents. This review article is based on current studies conducted in the last four years, evaluating the effectiveness of food-based natural antimicrobials in food, agriculture, medicine, and pharmacology.

## 1. Introduction

Antimicrobial agents are widely used to control microbial growth in process and products in industry. They are employed for manufacturing or preservation purposes in many areas, including but not limited to medicine, food, cosmetics, textiles, and nanotechnology. However, they have triggered concerns about potential side effects on human health and the environment or development of antibiotic resistance. In addition, synthetic chemicals in the food industry are also officially regulated due to their excessive consumption and have been prohibited in certain countries [1]. Therefore, there is an increasing trend for natural antimicrobial agents. Natural plant-derived antimicrobials, such as phenolics and organic acids, have been increasingly reported as promising alternatives to conventional chemical antibacterial agents owing to their safety [2].

Food processing wastes are generated in large amounts such as peels, pulps, seeds, husks, or kernels. These wastes generally accumulate in landfills, which have negative environmental impacts due to microbial degradation [3,4,5]. They are important sources of phytochemical compounds such as phenolics, carotenoids, essential oils, terpenoids, and dietary fiber [6,7,8,9,10]. These phytochemicals are extracted from the food processing wastes through different extraction techniques [11]. Novel extraction methods, including ultrasound-assisted extraction [12,13], microwave-assisted extraction [14], and pressurized liquid extraction [15], are found to be efficient methods to extract valuable bio-molecules from food waste. Therefore, most of this wasted food can be utilized as extraction substrate to recover large amounts of bioactive phytochemicals that can be re-included in the production process [16]. For instance, the use of tropical fruits and vegetable waste as a source of natural antimicrobials against food-borne pathogenic bacteria was reported [17]. The extract of grape industry by-product inhibited pathogenic food-borne bacteria [18] and bioactive phytochemicals, antioxidant properties, and antimicrobial activity of banana peel extract were determined [19]. Moreover, in addition to their antimicrobial activities, food waste rich in phytochemicals can be used in production of value-added products, food additives, therapeutic agents, or many industrially important products due to their antioxidant, antimicrobial, therapeutic, and nutritional attributes [20].

**Figure 1:**
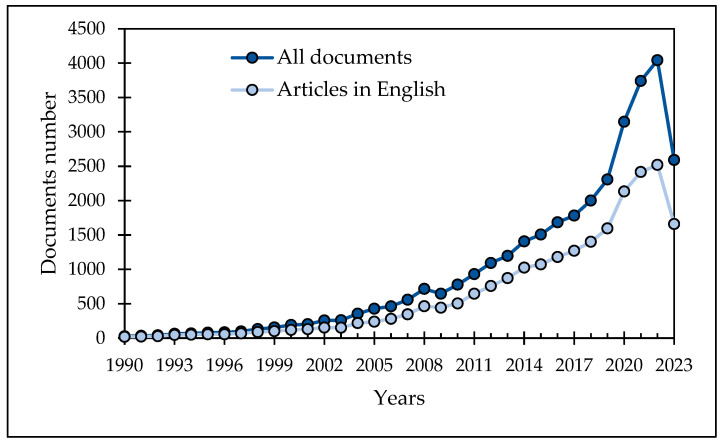
Document numbers on natural antimicrobials indexed in Scopus since 1990.

**Figure 2:**
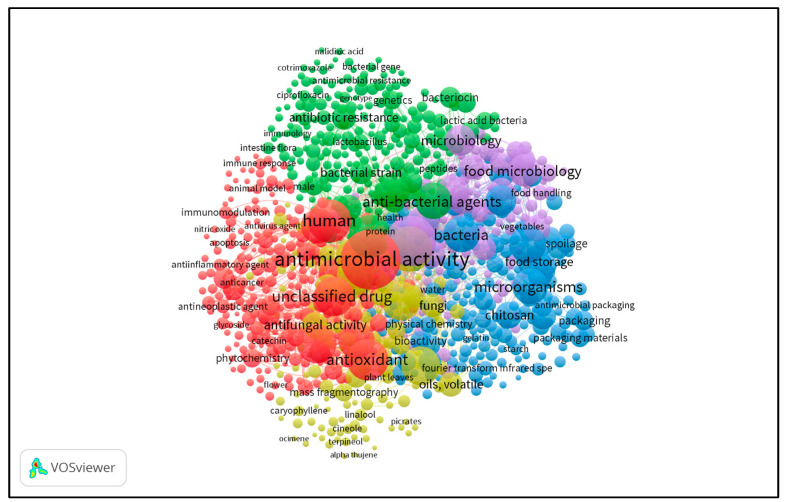
The bibliometric map of natural antimicrobials since 2020.

Research articles (23,030) and reviews (6625) were the most common document types published for “natural antimicrobials” of the 33,054 manuscripts (Figure 1). The number of articles published in English is 22,134, and approximately 40% of these articles have been published in the last four years (2020–2023). Therefore, this review focused on the articles published in the last 4 years, and the VOS Viewer program was applied to visualize the research on natural antimicrobials and create a bibliographic map (Figure 2). The present study reviews recent advancements in the antimicrobial use of food wastes in food, agriculture, medicine, and textile industries. It is necessary to conduct further research to explore cost-effective production methods of natural antimicrobials and their novel applications in industry. This policy would enhance the value of industrial products, create novel markets, and reduce the environmental impact of food waste accumulation in landfills.

## 2. Food Waste Composition

Based on the Food and Agriculture Organization of the United Nations (FAO) descriptions, food waste essentially refers to food with reduction in quantity or quality during retailers’ processing, food service providing, and people consuming. In general, the main sources of food waste are human food waste, industrial food waste, domestic food waste, and animal slaughtering waste but the composition of each group may be varied based on the time, area, processing, and human nutritional habits. Remarkably, fruit and vegetable substances account for 85% of the total food waste and the main organic materials of them are carbohydrate, proteins, and lipids [21,22]. According to the data provided by the FAO, it has been approximated that around 1.6 billion metric tons of food go to waste annually, out of which 1.3 billion metric tons consist of edible food substances, portions, or products. Around 30% of all food production each year, amounting to an estimated quantity, has the potential to provide sustenance for approximately 1.26 billion people suffering from hunger [20,21]. Large quantities of food waste not only impact humans, but also disrupt the delicate balance of various environmental ecosystems and discharge carbon dioxide, giving rise to global concerns [21]. However, from another point of view, food waste is a valuable source of beneficial compounds such as vitamins, minerals, prebiotics, pectin, fiber, and polyphenols that can be valorized to value-added products [23]. At present, there exist multiple technologies that can be employed to make use of food waste effectively. These technologies include composting food waste, creating value-added products, and producing biogas. These methods are adopted as means to valorise food waste and create novel economic opportunities [22].

According to the food waste categories, each group of food waste includes various items. For instance, the human food waste group contains many cereals and pulses such as rice, wheat, buckwheat, barley, corn, millet, and sorghum, the processing of which results in brans, husks, stems, leaves, etc. These kinds of food wastes can be utilized as fertilizer, building materials, or additive fiber. In addition to cereals, fruits and vegetables also contain high amounts of essential nutrients, water, carbohydrates, minerals, fiber, vitamins, and polyphenolic compounds and can be considered as food waste at any time from main sources to the end of the food supply chain [20,24]. To address the negative impact of food waste, innovative strategies such as microbial fermentation, production of single-cell proteins (SCPs) and single-cell oils, as well as enzyme and volatile fatty acid production offer promising alternatives compared to traditional methods such as landfilling [20].

Another important category of food waste refers to industrial food waste such as dairy and edible oil by-products. Dairy food waste is not only rich in carbohydrates, lipids, and proteins, but also contains high level of nitrogen. This composition of dairy food waste makes it possible for them to undergo microbial-mediated processing in order to produce value-added materials such as ethanol, SCPs, or prebiotics [25]. In the edible oil industry, oilseed cakes and meals, by-products of edible oil processing, are suitable sources of protein that are utilized for the production of animal feedstocks and fertilizer. Moreover, the effluent of the edible oil industry is an important hazardous food waste that requires serious attention to prevent environmental problems. Innovative biological approaches using microorganisms are employed to degrade organic compounds existing in the oily effluents and produce value-added materials such as biosurfactants. Other types of edible oil waste such as sterols, tocopherols, and squalenes are used in pharmaceutical and cosmetic industries [26,27].

Various types of food are produced in the livestock industry considered as the main portion of food supply chain. Accordingly, this industry produces huge amounts of animal by-products and wastes such as hair, skin, horns, blood, fat, soft tissue, bones, deboned leftovers, and feathers [28]. These by-products are subjected to being used for the production of biogas, probiotics, blood-containing foods, biodiesels, etc. [29]. Along with the livestock industry, the seafood industry can produce large quantities of food waste, including uneatable portions of fish, shrimp, lobster, and crab shells, scales, and endoskeletons. Valuable compounds in these wastes include chitin, chitosan, glycosaminoglycan, astaxanthin, calcium carbonate, and proteins, some of which, such as chitosan and glycosaminoglycan, showed antimicrobial activities. However, pathogens, toxins, and other hazardous materials may be present in seafood wastes, which limits their extensive usage for various purposes [30,31].

Having considered valuable materials existing in different food wastes, there is significant potential to promote sustainability and a circular economy by reducing the amount of food waste, producing value-added products, and extracting applicable materials such as bioactive compounds from food waste. Polyphenols, proteins, vitamins, minerals, organic acids, polysaccharides, lipids, and carbohydrates are considered as food waste bioactive compounds. These health-promoting materials, particularly polyphenols, are frequently found in plant-based foods such as fruits, tea leaves, and coffee grains [20]. For example, apple peel contains substantial amounts of flavonoids with antioxidant activity [32]. Occasionally, bioactive compounds of food waste reveal antimicrobial activities against eukaryotic parasites and pathogenic bacteria. For instance, bioactive compounds of banana have inhibitory effects on Leishmania [33]. Other antimicrobial agents found in food waste include peptides, phenolic compounds, terpenoids, and polysaccharides [34]. These agents can be used as food additives and preservatives to increase products’ shelf life [20].

## 3. Application of Natural Antimicrobials

### 3.1. Application of Natural Antimicrobials in Food Products

In the food industry, increasing the nutritional value, preventing microbial spoilage, and extending the shelf life of food products are of great importance [35]. For this purpose, interest in natural antimicrobials, which are alternatives to the use of synthetic chemicals generally preferred, is increasing day by day [36]. Considering the use of food processing by-products (e.g., olive oil mill wastewater), fruit and vegetable products (peels, seed, and pomace), various herbs, spices, and flowers (essential oils) are considered as natural antimicrobials with their bioactive compounds [37,38,39,40]. Extracts and powders of these plant-based products, which show naturally antimicrobial properties, have been evaluated both to increase the quality of bakery, meat, and dairy products and to extend their shelf life (Table 1). Natural antimicrobials can also enhance the nutritional value of food products; however, the use of natural antimicrobials in the food industry requires consideration of several factors such as effectiveness and sensory impact.

- Bakery products

Extracts or powders derived from fruit and vegetable peels and pomace can be used as additives in baking and pastry applications due to their antimicrobial and antioxidant properties.

In a conducted study, extracts from the peels of both sweet orange (*Citrus sinensis*) and pomegranate (*Punica granatum*) exhibited antimicrobial effects against *Pseudomonas aeruginosa*, *Serratia marcescens*, *Escherichia coli*, *Staphylococcus aureus*, *Bacillus subtilis*, *B. cereus*, and *Klebsiella pneumoniae* [41]. Additionally, recent research highlighted the substantial phytochemical content present in orange and pomegranate peels, with limonene and punicalagin being their prominent constituents. When utilized as additives in cakes, these peels were found to not only prolong the shelf life of the cakes, but also hinder microbial spoilage, as demonstrated in a recent study [41].

In a study exploring the viability of incorporating banana peel powder as a cake additive, the analysis concluded that cakes formulated with banana (*Musa* spp.) peel powder (12%) exhibited superior sensory attributes in terms of taste, odor, mouthfeel, crust color, and overall quality compared to the control cake [42]. Additionally, the addition of banana peel powder at a rate of 16% enriched the cakes with essential minerals (phosphorus, calcium, potassium, and iron), showcased notable antioxidant properties, and effectively decreased the overall microbial load. Likewise, papaya (*Carica papaya*) seed powder and the oil extracted from processed papaya fruit were also evaluated for cupcake production [43]. Notably, the inclusion of 15% papaya powder resulted in improvement levels of protein (16.89%) and fiber (3.28) content of the cupcake the inclusion of 15% papaya powder. It was also observed that the powder and oil of papaya (major components are oleic acid, stearic acid, palmitic acid, benzyl isothiocyanate, linoleic acid, and 4-Hydroxybenzoic acid) exhibited antimicrobial activities against microorganisms such as *B. subtilis*, *S. aureus*, *Enterococcus faecalis*, *P. aeruginosa*, *B. cereus*, *Salmonella* spp., *Candida albicans*, and *Aspergillus* spp. [43]. On the other hand, Caleja et al. [55] suggested that chestnut (*C. sativa*) flower extract can be used as an alternative to potassium sorbate to obtain “pastel de nata”, a Portuguese dessert. In addition, extracts of fruits and flowers can be used as a natural dyestuff in cake production due to their natural color properties [56,57].

- Meat products

Microbial spoilage is a major concern in animal food products such as meat and poultry [58]. To delay microbial spoilage and extend the shelf life of animal-based products, the use of natural antimicrobial compounds derived from food waste is proposed as a strategy according to different studies [44,45,46,47,48,49,50].

The effects of extracts and powders obtained from quercetin-rich yellow and red onion (*Allium cepa* L.) skins on beef burgers were investigated and it was found that these extracts and powders increased the antioxidant activities, total phenolic, and total flavonoid contents of the burger [44]. In addition, it was determined that these extracts did not cause any sensory changes in beef burgers and no significant increase in microbial load was observed in meat samples stored for 15 days. Therefore, it is suggested that these natural antimicrobial extracts can be used as additives or preservatives in beef burgers due to their antioxidant and antimicrobial effects [44]. In another study in which ethanolic extracts of red onion skins (0.1%) were applied to refrigerated beef patties, it was reported that they reduced microbial contamination and extended the shelf life of meatballs up to 9 days [45].

Lemon (*Citrus limon* (L.) Osbeck) and orange peels improve microbial and chemical values and improve sensory properties when applied to raw chilled minced beef [46]. However, although the use of high amounts of lemon and orange peel powders (10%) reduced the microbial load, it adversely affected sensory properties. Therefore, it is recommended to add 5% lemon peel powder to the processed meat to both reduce the microbial load and not affect the sensory analysis [46].

It has been determined that pomegranate peel extract has high phenolic (total phenolics content, 149.75 mg/g) and flavonoid (total phenolics content, 13.13 mg quercetin equivalents/g) contents and exhibits antioxidant activity from Ganesh variety [47]. The microbial load of the buffalo meat sample without the extract exceeded the spoilage limit (10^7^ cfu/g) after 12 days, while the meat samples containing the pomegranate extracts (1.0 and 1.5%) exceeded this limit at 20 days. The bioactive compounds in pomegranate peel are suggested to help preserve buffalo meat during refrigeration, thereby reducing fruit peel waste and environmental pollution [47]. Similarly, Hadab and Dakheel [48] suggested that solutions obtained from pomegranate pomace can be considered as natural preservatives of meat products even at low concentrations. Except for pomegranate extracts, potato peel powder and apple peel powder extracts were also found to significantly reduce total bacterial growth in chicken meatballs [49]. In addition, the effects of lemon, orange, grapefruit (*Citrus paradisi*), and banana peel powders on the oxidative stability, microbial quality, physicochemical properties, and sensory properties of chicken patties were investigated and it was found that lemon peel powder contained high levels of phenolic (90.5 mg gallic acid/g) and flavonoid (35 mg rutin/g) compounds and exhibited high antioxidant activity [50]. The addition of banana peel powder to chicken meatballs caused an increase in the amount of protein (22.18 g/100 g) and a decrease in the amount of fat (10.52 g/100 g). In addition, it was observed that chicken patties to which fruit peel powder (1%) was added had a significantly lower microbial load than the control group [50]. Similar to pomegranate extracts, cranberry (*Vaccinium* subg. *Oxycoccus*) and black chokeberry (*Aronia melanocarpa*) extracts effectively inhibit *S. aureus*, *E. coli*, and *Streptococcus pyogenes* that cause spoilage during raw pork meatball production [59,60,61].

- Dairy products

Food-based natural compounds, especially extracts of fruits, vegetables, herbs, and spices, can be used to control fermentations and foods with a high risk of contamination (e.g., ready-to-eat and dairy products) as they are defined as antimicrobial [62,63,64,65]. Dairy products are susceptible to contamination and, therefore, pose a risk to consumer health due to their relatively short shelf life [66]. Microbial spoilage in these products can be delayed by using food-based extracts because of their phytochemical contents and antioxidant and antimicrobial activities [51,52,53,54].

El-Kholany et al. [51] aimed to evaluate the effect of lemon peel extract, obtained using distilled water, on labneh cheese, on the physical properties, microbiological properties, and sensory qualities of the cheese. While the total bacterial count reached its maximum after 7 days of storage in the control labneh, the labneh cheese with added lemon extract showed no detection of psychrophilic bacteria, yeast, mold, and coliform bacteria for up to 3 weeks. The extract exhibited antimicrobial activities against *B. cereus*, *S. aureus*, *Listeria monocytogenes*, *B. subtilis*, *E. coli*, *S. typhimurium*, *P. aeruginosa*, *C. albicans*, and *A. fumigatus*. Furthermore, the addition of lemon peel extract up to 4% was found to have a positive effect on the flavor of labneh cheese [51]. In another study, in which the extract obtained using pomegranate pomace was added to strawberry yogurt smoothies, the extract inhibited the growth of pathogenic bacteria and fungi such as *L. monocytogenes*, *P. aeruginosa*, *K. pneumoniae*, *A. niger*, and *C. glabrata*, and the extract-added smoothies exhibited lower microbial load compared to control samples. Moreover, the addition of pomegranate peel extract improved the color, texture, and quality of the smoothies [52]. Similarly, the addition of pomegranate peel extract to traditional butter showed lower levels of microbial populations, suggesting that pomegranate extracts can be used as a natural antioxidant and antimicrobial source for preserving traditional butter [54]. In a different study examining avocado peel extract as a preservative for mayonnaise, the extract showed antimicrobial activity against *S. aureus*, *S. epidermidis*, and *E. coli*, and no change in chemical and physical properties was observed in mayonnaises to which 0.5% and 1% extracts were added compared to the control group. Avocado (*Persea americana*) peel extract has shown similar performance to synthetic antioxidants and has been suggested to have the potential to be a natural antimicrobial agent [53].

It was determined that the antimicrobial activities as well as qualities of dairy products (yogurt, cheese, and butter) were improved by adding essential oils and extracts of herbs and spices [66]. Yuningtyas et al. [67] reported that yogurts with arrowroot (*Maranta arundinacea*) added both contain higher lactic acid bacteria and have higher antioxidant activity. Shirani et al. [68] also mentioned that *Echinops setifer* extracts improve yogurt quality and functionality by containing natural bioactive sources. Maiza et al. [69] reported that ghee (butter oil) samples flavored with aromatic plants contain potential therapeutic components and can improve skin regeneration. Afiyah et al. [70] evaluated mango (*Mangifera indica*) extracts in the production of yogurt and reported that it has inhibitory activity against *S. aureus*, *E. coli*, and *B. cereus* in populations lower than 5.00 log cfu/mL. Selahvarzi et al. [71] evaluated orange peel and pomegranate peel extracts as natural preservatives in functional beverages and reported that the extracts showed antibacterial activity against *S. aureus* and *E. coli*. In addition, it has been reported that orange peel extracts can be a suitable natural preservative regarding sensory acceptance [71].

**Figure 3:**
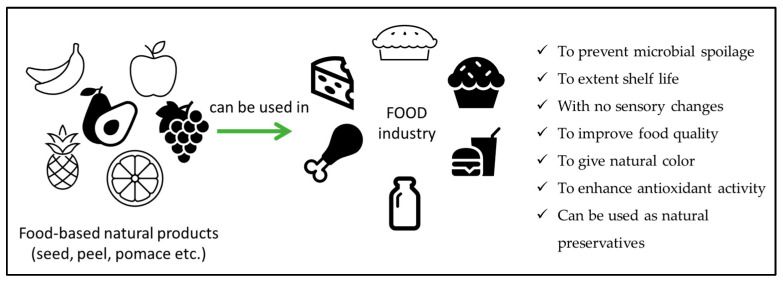
The applications of food-based natural products in the food industry.

In general, the discovery of natural antimicrobials as food preservatives offers a promising approach to enhance food safety and extend shelf life (Table 1; Figure 3). Further research and development in this field will contribute to the effective use of natural antimicrobials in the food industry.

### 3.2. Application of Natural Antimicrobials in Agriculture and Livestock

Natural antimicrobials have been demonstrated in a variety of applications in agriculture. The utilization of agro-food waste extracts, which possess antimicrobial properties and show great potential as biofungicides, biopesticides, bioinsecticides, and biostimulators, and their applications beyond plant-based systems are discussed in this section.

Phytopathogenic fungi play a significant role in plant diseases [72]. The use of plant-based food waste extracts or powders with antifungal properties as natural biofungicides is being investigated for their potential in disease management [73,74,75]. Endophytic microbes have been reported, including the species of *Trichoderma* sp., *Aspergillus* sp., *Fusarium* sp., *Mucor* sp., *Aeromonas* sp., *Corynebacterium* sp., *Enterobacter* sp., *Sphingomonas* sp., and *Penicillium* sp., and *Bacillus* sp. [73,76]. Pesticide application was observed to influence the endophytic microbial population [77]. A study examined the effect of *Archidendron pauciflorum* (jengkol) peel powder to prevent Moler disease in onions [73]. The effect of jengkol peel powder on preventing Moler disease in onions was investigated in a study. The microbial diversity in onions treated with plant-based pesticides and untreated onions showed a moderate level [73]. In another study, the effects of jengkol peel extract were investigated against *Curvularia* sp., the causative agent of leaf spot disease in oil palm seedlings [74]. The results indicated that, when jengkol peel extract was applied at a concentration of 50%, it led to a 36% reduction in the fungal colony diameter. Additionally, at a concentration of 30%, the extract completely inhibited the growth of the fungal colony. These findings highlight the significant antifungal activity of jengkol peel extract against *Curvularia* fungal colonies. This suggests the potential application of the extract as a biofungicide for controlling the disease [74]. Teixeira et al. [75] demonstrated the antifungal potential of garlic (*Allium sativum*) peel extract, showcasing its effectiveness against a range of phytopathogenic fungi. Notably, ex situ experiments conducted with apples revealed that the application of garlic peel extract led to a significant reduction in lesion size caused by *Colletotrichum acutatum* spores. Further investigations in this study have shed light on the antifungal mechanism of action, indicating that the extract targets the fungal plasma membrane and cell wall. These insights underscore the promising role of garlic peel extract as a viable alternative biofungicide in agricultural contexts [75].

The synthesis of metal nanoparticles has emerged as a recent trend in green chemistry [78]. In a particular study, pomelo *(Citrus maxima*) fruit juice and peel extract were used to synthesize silver nanoparticles (AgNPs). Remarkably, the AgNPs derived from pomelo exhibited antimicrobial properties against *S. aureus* and *K. pneumoniae*. Additionally, nano-primed seeds of *Zea mays*, *Glycine max*, and *Cicer arientum* exhibited enhanced germination rates and augmented plant growth in comparison to the control group. This research underscores the potential of repurposing plant-based food waste without commercial value for nanoparticle synthesis, with subsequent applications in the agricultural sector [78].

In a study, essential oils were extracted from grapefruit peel using both microwave-assisted hydrodistillation and conventional hydrodistillation methods. The investigation unveiled that the procured essential oils displayed notable antimicrobial effects against *E. coli*, *P. aeruginosa*, *S. aureus*, and *C. albicans*. Additionally, these essential oils exhibited toxic properties against adult *Ceratitis capitata*, a notorious fruit pest, with LD50 at 9.12 µL EO/acetone and LD90 at 13.18 µL EO/acetone [79]. This research highlights the potential utility of grapefruit-peel-derived essential oils, enriched with natural antimicrobial agents, as bioinsecticides.

Moreover, the scope of natural antimicrobial applications extends beyond plant-based uses, encompassing incorporation into animal feed to proficiently combat pathogenic bacteria accountable for animal diseases [80]. In a study, peanut shells were investigated as an antibacterial food additive in poultry diets to prevent the proliferation of *Salmonella*. The outcomes of the study underscore the conceivable utility of peanut (*Arachis hypogaea*) shells as an antimicrobial supplement for animal feed. This investigation contributes to establishing the value-enhanced utilization of peanut shells or extracts as natural antimicrobial additives in the context of chicken production [80].

Natural antimicrobials possess a wide range of applications in agriculture, including managing animal health, battling plant diseases, and enhancing plant growth. Ongoing research and development in this field has the potential to further expand the applications of natural antimicrobials in agriculture, contributing to sustainable and safe food production systems.

### 3.3. Application of Natural Antimicrobials in Medicine and Pharmacology

Food-based natural antimicrobials have attracted considerable interest within the domains of medicine and pharmacology, primarily owing to their potential therapeutic uses (Table 2). These antimicrobials provide an alternative to traditional antibiotics and pharmaceuticals, further augmented by their advantage of fewer side effects.

Natural phytochemicals present in garlic and turmeric (*Curcuma longa*), such as allicin and curcumin, have gained attention for their antimicrobial potential [81]. These compounds offer various health benefits, especially against multidrug-resistant pathogens. These phytochemicals inhibit enzymes and efflux pumps, providing an alternative to antibiotics with fewer side effects. Their effectiveness extends to addressing bacterial, viral, and fungal infections. The utilization of nanoformulations further heightens their safety and efficacy in the management of microbial infections [81].

Furthermore, medical-grade honey (MGH) shows promise as a therapy for challenging biofilms in the wound healing [82]. Among the tested MGH-based formulations in a study conducted by [82], L-Mesitran Soft exhibited the strongest antimicrobial activity against biofilms, particularly those formed by *P. aeruginosa*. The synergistic effects of its components, including raw MGH, vitamins C and E, and other ingredients, contribute to its efficacy [82]. Manuka honey has unique chemical constituents that contribute to its health benefits [83,84]. It has potential as an alternative remedy for infections, wound healing, and cartilage repair. It also shows chemo-protective and anticancer activities [84]. Propolis, a substance produced by honeybees, exhibits diverse clinical applications due to its composition variability [85]. It possesses antioxidant, anti-inflammatory, antimicrobial, anticancer, analgesic, antidepressant, anxiolytic, and immunomodulatory effects [86]. Traditionally used for wound healing and treating purulent disorders, propolis has gained research attention post-world wars. Recent studies highlight its potential in managing cancer, infections, and parasitic-related symptoms [85,86].

**Table 2:** Application of food-based natural antimicrobials in medicine and pharmacology.

Natural Antimicrobials	Applications	References
Chitosan, aloe vera, and olive leaf extract	Surgical site infections	[87]
Lactobacillus	Cardiovascular-related diseases	[88]
Plant-based nanoparticles	Nanotherapeutic drugs with antimicrobial properties	[89]
Andrographis paniculate	Infectious disease	[90]
Nanomaterials & bacteriocins	Therapeutic strategies	[91]
Essential Oils	Antimicrobial, Antiviral	[92]
Different antimicrobials from nature	Peroxidase-catalyzed systems	[93]
*Nigella sativa* L.	Therapeutic use	[94]
Aloe vera, Angelica gigas, Astragalus membranaceus, Ganoderma lucidum, Panax ginseng, Scutellaria baicalensis	Coronavirus disease	[95]
Natural volatiles	Food-based natural antimicrobials	[96]
Phyllanthus emblica	Vitamin C, minerals, amino acids source	[97]
European barberry, garlic, sweet potato, biter gourd	Effect on hypoglycemic	[98]
Garlic & turmeric	Antimicrobial application	[81]
Vernonia amygdalina	Hair shampoo	[99]
Bryophyllum pinnatum	Hair shampoo	[100]

Berberine exhibits diverse effects on enzymes, receptors, and cell signaling pathways, suggesting its potential as a therapeutic agent [101,102,103]. Despite limited bioavailability, emerging evidence implicates the gut microbiota in mediating its effects. Berberine shows promise in various pharmacological areas, including cancer, infections, inflammation, and hypoglycemia [103]. Structural modifications aim to improve its pharmacokinetics and therapeutic properties.

In pharmacological applications, natural antimicrobial compounds such as tea tree oil, neem oil, or saffron can be incorporated into creams or ointments for topical applications to treat various skin infections and dermatological conditions [104,105]. Saffron (*Crocus sativus*) petal extract, in particular, has shown potential as a natural antimicrobial source, offering an alternative to chemical preservatives in creams and contributing to the management of infectious diseases [105].

Tea tree oil derived from *Melaleuca alternifolia* has been shown to have various dental applications [106]. It has been found effective in managing Demodex-related diseases, reducing mite counts, relieving symptoms, and modulating the immune system. Its antiparasitic, antibacterial, antifungal, anti-inflammatory, and wound-healing properties make it a versatile treatment option [106,107]. Certain food-derived antimicrobials such as coconut oil have also been used in shampoos to combat dandruff, scalp infections, or lice infestations [108]. Their study formulated a liquid shampoo using neem-leave-infused oil, known for its antibacterial effects. The shampoos developed in the study met the required standards for properties such as smell, moisture content, and pH.

Food-based natural antimicrobials offer promising alternatives in medicine and pharmacology, with efficacy against multidrug-resistant pathogens and various infections. Examples include garlic, turmeric, honey, cranberry extracts, propolis, saffron, and coconut oil. However, further research and clinical studies are necessary to determine their effectiveness, safety, optimal dosage, and specific applications.

### 3.4. Application of Natural Antimicrobials in Polymers

The utilization of food-based natural antimicrobials in conjunction with polymers offers exciting possibilities for enhancing the quality and safety of food packaging materials, including wrapping papers and coatings (Table 3). By incorporating natural antimicrobials, such as essential oils, and natural extracts into polymers, food packaging can significantly extend the shelf life of packaged products, reduce food waste, and enhance food safety [109]. Some commonly used antimicrobials in food packaging include essential oils, bacteriocins, chitosan, and grapefruit seed extracts [110]. Studies have shown that layered double hydroxides, particularly zinc and aluminum hydroxides, are effective against bacteria such as *E. coli* and *S. aureus* [110].

Packaging materials coated or infused with natural antimicrobials help maintain the nutritional quality of perishable foods. Essential oils, polysaccharides, polypeptides, and enzymes exhibit antimicrobial activity against bacteria and fungi, thereby extending the shelf life of products and inhibiting the growth of spoilage and pathogenic microorganisms [121]. Studies have demonstrated that the food-based natural products are effective against pathogens such as *S. aureus*, *L. monocytogenes*, and *E. coli* in cheese [122].

Extending the shelf life of products is another notable benefit of using natural antimicrobials in food packaging. The antimicrobial properties of food-based natural antimicrobials can inhibit the growth of microorganisms on the surface of food packaging materials. This prevents microbial contamination and extends the shelf life of packaged products, reducing the need for chemical preservatives. Effective management of food spoilage can be achieved through the use of non-synthetic chemicals, nanomaterials, active packaging, and decision support systems [124]. Additionally, minimizing damage during handling, exploring alternative food sources, and implementing efficient storage practices contribute to a safer and more sustainable food environment [119].

Incorporating natural antimicrobials minimizes or eliminates the need for synthetic antimicrobial additives or chemical preservatives. This aligns with the increasing consumer demand for clean-label products and promotes a more natural and sustainable approach to food packaging [115]. Natural antimicrobials and nanoemulsion (NEs) formulation ingredients are of particular interest, as consumers prefer naturally processed foods. While there is substantial research in this field, there is a lack of investigations into the application of antimicrobial-loaded NEs in real food systems [115].

The use of food-based natural antimicrobials in food packaging aligns with the growing focus on sustainable and eco-friendly practices. These antimicrobials are derived from renewable sources, reducing the environmental impact associated with conventional packaging materials and chemical additives. However, challenges exist, including their potential toxicity to human cells and the difficulties of large-scale production due to cost and energy requirements [120]. More references and applications are presented in Table 1.

It is essential to note that incorporating natural antimicrobials into polymer matrices for packaging applications requires careful consideration of factors such as compatibility, migration potential, stability, and regulatory compliance. Further research and development are necessary to optimize formulations, evaluate long-term effectiveness, assess potential interactions with packaged foods, and ensure the safety of these antimicrobial packaging materials.

### 3.5. Application of Natural Antimicrobials in Textile

Agricultural and food industry processing wastes and/or by-products can be utilized for dying following extraction processes for textile purposes [125,126]. Hosseinnezhad et al. [127] suggested that two natural tannin-rich mordants (chebulagic acid, gallic acid, chebulinic acid, and ellagic acid), yellow and black myrobalan (*Terminalia chebula* and. *T. citrina*), could be a natural procedure for the production of green Iranian carpet. Adeel et al. [128], on the other hand, claimed that the natural dye obtained from coffee can have a good impact on the ecosystem and global society, since it is not carcinogenic. Moreover, Samant et al. [129] reported that dyed fabric obtained from *Acacia catechu* showed antibacterial activities against *E. coli* and *S. aureus*. The pigments extracted from food industry wastes have antibacterial and antifungal activity for use in textile and as wound bandages in medicine [130,131,132,133]. Hakim et al. [134] suggested that the pigment obtained from *Senegalia catechu* can be applied on various foods such as apples and cookies and, therefore, can be used as an edible ink. Similarly, betalains extracted from red pitaya (*Hylocereus costaricensis*) can be used as a bio-based dye for food packaging or plant-based edible cups [135].

Sar and Akbas [131] suggested that bacterial cellulose produced from olive oil mill wastewater can be used as a biomaterial in the textile industry, since it has the appearance of brown leather and old paper textures. Bacterial cellulose produced from food waste can be modified with antimicrobial molecules such as bioactive compounds, nanoparticles, antibiotics, etc., to gain antimicrobial properties [136,137]. For this purpose, strong antibacterial activity can be gained against various bacteria such as *S. aureus* and *E. coli* by ex situ modification of bacterial cellulose produced from food wastes with various plant extracts (*Euclea schimperi*, *Anogeissus dhofarica*, and *Withania somnifera*) [138,139]. On the other hand, Zhang et al. [140] reported that bacterial cellulose modified with tannic acid and magnesium ions had strong antibacterial activity against *S. aureus*, *E. coli*, and *P. aeruginosa* and reduced biofilm formation of *S. aureus* and *P. aeruginosa* by 80%. Similarly, Shen et al. [141] modified bacterial cellulose with chitosan and ferulic acid, showing strong antibacterial activity against *S. aureus* and *E. coli*. The modified bacterial cellulose can be used as food packaging and wound-healing materials [140,141].

### 3.6. Application of Natural Antimicrobials in Nanotechnology

Combinations of natural antimicrobials with nanotechnological strategies are carried out to improve antimicrobial activity in the use of food surface disinfection or food packaging (Table 4) [142,143,144].

Grape (*Vitis* sp.) seed oil is a potential antimicrobial and contains flavonoids, tocopherols, and other antimicrobial compounds. Arumugam et al. [149] developed a polybutylene adipate terephthalate (PBAT) biocomposite film by combining grape seed essential oil with silica nanoparticles (SiO_2_ NPs). It has been stated that the developed new material has increased the antimicrobial activity and can be used in active food packaging applications. Similarly, Alizadeh Sani et al. [150] developed a nanocomposite film using natural pigments and titanium dioxide nanoparticles (TiO_2_ NPs). The developed film showed both antimicrobial and antioxidant activities. Song et al. [146] determined that mandarin (*Citrus reticulata*) essential oil incorporated into chitosan nanoparticles damaged the cell membranes of *S. aureus* and *E. coli* and there were changes in cell morphology. It has been suggested that the developed product inhibits the formation of biofilm and destroys the mature biofilm, and it can be used for the preservation of pork [146]. In a similar study, it was determined that the films developed with berberine-cinnamic acid nanoparticles inhibited microbial growth in chickens and extended the shelf life of food products [148].

On the other hand, it was suggested that the mixture of cinnamon oil and silver nanoparticles showed antimicrobial and antibiofilm activity against *Streptococcus agalactiae* isolated from milk from clinical mastitis and, therefore, nanoparticles loaded with natural-based antimicrobials could be used in the treatment of dairy farms against mastitis [151].

Gupta et al. [147] produced silver nanoparticles using curcumin, a wound-healing agent, with hydroxypropyl-*β*-cyclodextrin complex and loaded them into a bacterial cellulose hydrogel with moist wound-healing properties. This developed product exhibited antimicrobial activity against *S. aureus*, *P. aeruginosa*, and *C. auris* [147]. Donga et al. [152], on the other hand, evaluated mango seed to produce gold nanoparticles for their antimicrobial and cytotoxic potential and suggested that food waste used in nanoparticle synthesis could be considered as a source of anticancer agents.

## 4. Conclusions

In conclusion, the enormous various fruit and vegetable wastes are potential sources of antioxidants and antimicrobial agents. These valuable bioactive compounds recovered from food waste have important applications in food, agriculture, medicine, and pharmacology industries.

In the food industry, the use of food-based natural antimicrobials plays a crucial role in processed foods that are highly susceptible to microbial spoilage and have a limited shelf life. These antimicrobials not only enhance the nutritional quality of foods by enriching them with antioxidants, but also effectively prevent microbial contamination, leading to extended shelf life. In addition, the inclusion of natural antimicrobials in food packaging contributes to extending the shelf life of meat products, thereby increasing their preservation and safety.

In agricultural applications, these natural antimicrobials act as environmentally friendly pesticides by eliminating phytopathogenic microbes that cause plant diseases. Such sustainable approaches have the potential to revolutionize agriculture while reducing reliance on traditional chemical pesticides.

In medicine and pharmacology, the investigation of these natural antimicrobials as potential therapeutic agents highlights their importance in the development of new drugs and treatments.

The overall review article provides a comprehensive review of current studies conducted over the past four years. The use of these bioactive compounds not only offers sustainable solutions for waste management, but also shows promise in addressing various challenges associated with the development of therapeutic interventions for food safety, agricultural sustainability, and human health.

## Figures and Tables

**Table 1:** Studies investigating the potential of using different wastes as food additives and/or preservatives.

Food	Products	Material	Outcomes	References
Bakery products	Extract	Orange peel Pomegranate peel	Antimicrobial affects against *P. aeruginosa*, *S. marcescens*, *E. coli*, *S. aureus*, *B. subtilis*, *B. cereus*, and *K. pneumonia.* Extend the shelf life of cakes and delay microbial spoilage.	[41]
	Powder	Banana peel	Improvement in sensory characteristics, increased mineral content, and antimicrobial and antioxidant activity when added to cake.	[42]
	Powder or seed oil	Papaya seed	Antimicrobial activity against *B. subtilis*, *S. aureus*, *E. faecalis*, *P. aeruginosa*, *B. cereus*, *Salmonella* spp., *C. albicans*, and *Aspergillus* spp. Enhance the flavor and nutritional value when added to chocolate cakes.	[43]
Meat products	Peels or extracts	Onion peels	Increased the total phenolic and flavonoid content and delayed microbial spoilage when added to meat.	[44]
	Extract	Onion peels	Reduced microbial spoilage, antioxidant, and antimicrobial activity, and extended shelf life of beef meatballs.	[45]
	Powder	Lemon peels Orange peels	Improved microbial and chemical values, enhance sensory characteristics when added to meat.	[46]
	Extract	Pomegranate peel	Reduced total colony count when added to buffalo meat.	[47]
	Pomace	Pomegranate pomace	Positive effects on total bacterial count, lipid oxidation, pH values, and extended shelf life of beef.	[48]
	Extract	Pomegranate peel Potato peel Apple peel	Reduced total bacterial growth when added to chicken meatballs. In addition, showed antioxidant properties	[49]
	Powder	Lemon peel Orange peel Grapefruit peel Banana peel	Lower microbial loads when added to chicken meatballs.	[50]
Dairy products	Extract	Lemon peel	Antimicrobial activity *against B. cereus*, *S. aureus*, *L. monocytogenes*, *B. subtilis*, *E. coli*, *S. typhimurium*, *P. aeruginosa*, *C. albicans*, and *A. fumigatus*. Extended shelf life and reduced microbial contamination	[51]
	Extract	Pomegranate pomace	Antimicrobial activity against *L. monocytogenes*, *P. aeruginosa*, *K. pneumoniae*, *A. niger*, and *C. glabrata.* Improvement in color, texture, quality, and lower antimicrobial load when added to smoothies.	[52]
	Extract	Avocado peel	Antimicrobial activity against *S. aureus*, *S. epidermidis*, and *E. coli*. When added, chemical and physical properties did not change the mayonnaise.	[53]
	Extract	Pomegranate peel	Lower microbial load when added to butter.	[54]

**Table 3:** Application of food-based natural antimicrobials with polymers.

Use	Natural Antimicrobials	References
Antimicrobial Packaging	Essential oils, bacteriocins, lysozyme, grapefruit seed extracts, chitosan.	[110]
	Starch, sugar beets, corn.	[111]
	Different natural antimicrobials.	[36]
	Biomass, microorganisms, Bio-based monomers.	[112]
	Plant-Derived, Animal-Derived, Microorganism-Derived.	[113]
	Pineapple, green tea, coconut, pecans, fenugreek seeds, mangoes, olive leaf, yellow onions, and soybean seeds.	[109]
	Carotenoids, tannins, alkaloids, anthocyanins, flavonoids, terpenoids, caffeic acid, and other organic acids.	[114]
Minimization of Synthetic Additives	Oils of eugenol, carvacrol, thymol, and basil.	[115]
	Peppermint, oregano, coriander	[116]
	Lemongrass oil	[117]
Extended Shelf Life	Rosemary, oregano, basil, and cedar essence.	[118]
	Bacteriocins, enzymes, essential oils, grapefruit seed extract, green tea extract, and cranberry extract	[119]
	Antimicrobial properties of carbon nanomaterials	[120]
	Bacteriocins	[121]
Preservation of Nutritional Quality	Lemongrass, garlic, cumin, green propolis, black cumin cedar, fennel, pennyroyal, and ginger	[122]
	Prosopis juliflora leaf	[123]
	Microparticles, microgels, nanoliposomes, nano micelles, and nanostructured lipid carriers generated from yeast.	[124]

**Table 4:** Application of food-based natural antimicrobials with nanoparticles.

Natural Antimicrobials	Application	Tested Microorganisms	Finding Results	References
Carvacrol	Encapsulation into liposomes or nanocapsules	*S. aureus* *Salmonella* *E. coli* *L. monocytogenes*	This can be used as a surface sanitizer	[142]
Thymol and/or carvacrol		*Salmonella*	Antimicrobial-loaded nanoliposomes displayed decreased anti-Salmonella activities.	[145]
Mandarin essential oil	Essential-oil-loaded chitosan nanoparticles	*S. aureus* and *E. coli*	Biofilm formation was inhibited, and mature biofilms were removed It can be used for pork preservation	[146]
Curcumin	Bacterial cellulose hydrogel containing silver nanoparticles using curcumin	*S. aureus*, *P. aeruginosa* and *C. auris*	It has potential wound-dressing application, and has broad-spectrum antimicrobial activity	[147]
Berberine-cinnamic acid	Nanocomposite films	*S. aureus* and *E. coli*	Microbial growth was inhibited, and food shelf life was extended	[148]

## Data Availability

Not applicable.
